# Potential Activities and Long Lifetimes of Organic Carbon-Degrading Extracellular Enzymes in Deep Subsurface Sediments of the Baltic Sea

**DOI:** 10.3389/fmicb.2021.702015

**Published:** 2021-09-17

**Authors:** Jenna M. Schmidt, Taylor M. Royalty, Karen G. Lloyd, Andrew D. Steen

**Affiliations:** ^1^Department of Earth and Planetary Sciences, College of Arts and Sciences, The University of Tennessee, Knoxville, TN, United States; ^2^Department of Microbiology, University of Tennessee – Knoxville, Knoxville, TN, United States

**Keywords:** extracellular enzymatic activity, Baltic Sea, International Ocean Discovery Program 347, peptidase activities, subsurface microbial ecosystem

## Abstract

Heterotrophic microorganisms in marine sediments produce extracellular enzymes to hydrolyze organic macromolecules, so their products can be transported inside the cell and used for energy and growth. Therefore, extracellular enzymes may mediate the fate of organic carbon in sediments. The Baltic Sea Basin is a primarily depositional environment with high potential for organic matter preservation. The potential activities of multiple organic carbon-degrading enzymes were measured in samples obtained by the International Ocean Discovery Program Expedition 347 from the Little Belt Strait, Denmark, core M0059C. Potential maximum hydrolysis rates (V_max_) were measured at depths down to 77.9mbsf for the following enzymes: alkaline phosphatase, β-d-xylosidase, β-d-cellobiohydrolase, N-acetyl-β-d-glucosaminidase, β-glucosidase, α-glucosidase, leucyl aminopeptidase, arginyl aminopeptidase, prolyl aminopeptidase, gingipain, and clostripain. Extracellular peptidase activities were detectable at depths shallower than 54.95mbsf, and alkaline phosphatase activity was detectable throughout the core, albeit against a relatively high activity in autoclaved sediments. β-glucosidase activities were detected above 30mbsf; however, activities of other glycosyl hydrolases (β-xylosidase, β-cellobiohydrolase, N-acetyl-β-glucosaminidase, and α-glucosidase) were generally indistinguishable from zero at all depths. These extracellular enzymes appear to be extremely stable: Among all enzymes, a median of 51.3% of enzyme activity was retained after autoclaving for an hour. We show that enzyme turnover times scale with the inverse of community metabolic rates, such that enzyme lifetimes in subsurface sediments, in which metabolic rates are very slow, are likely to be extraordinarily long. A back-of-the-envelope calculation suggests enzyme lifetimes are, at minimum, on the order of 230days, and may be substantially longer. These results lend empirical support to the hypothesis that a population of subsurface microbes persist by using extracellular enzymes to slowly metabolize old, highly degraded organic carbon.

## Introduction

Subsurface marine sediments contain a diverse community of heterotrophic microbes which metabolize organic carbon at extraordinarily slow rates (Hoehler and Jørgensen, 2013; Hoshino et al., 2020). There has been extensive research on the electron acceptors that subsurface heterotrophs use to gain energy, but the nature of electron donors that fuel these metabolisms is more mysterious (LaRowe et al., 2020). Heterotrophic microbes access large organic molecules *via* extracellular or periplasmic hydrolysis, which is catalyzed by extracellular enzymes (Benz and Bauer, 1988; Reintjes et al., 2017). These enzymes may be tethered to cell membranes or released freely into the extracellular medium; either of these strategies may be optimal depending on the situation (Vetter et al., 1998; Traving et al., 2015). Due to the extreme energy limitation and low concentrations of labile organic matter that characterize deeply buried marine sediments (Bradley et al., 2020), extracellular enzymes may be a particularly important bottleneck for energy acquisition: Enzymes are expensive to produce in terms of carbon, nitrogen, and energy, so it is important for extracellular enzymes to provide a positive “return on investment” to the microbes that synthesize them. At the same time, any specific enzyme typically catalyzes only a very narrow set of reactions, so extracellular enzymes produced by microbes must match the set of molecules that are present in the environment. Finally, interspecific interactions must be conducive to enzyme production: If “cheaters” (microbes which do not produce extracellular enzymes, but which metabolize hydrolysis products) can outcompete enzyme producers, this can in principle lead to the extinction of enzyme producers (Allison, 2005). In a system, such as subsurface sediments, in which immigration and evolution are slow (Walsh et al., 2016; Starnawski et al., 2017; Marshall et al., 2019), such an extinction could be permanent. Therefore, several factors suggest that metabolism of high molecular weight organic substrates is a more challenging proposition in the subsurface than in surface environments.

Nevertheless, active extracellular enzymes have been observed in a wide range of subsurface environments (e.g., Coolen and Overmann, 2000; Engelen et al., 2008; Lloyd et al., 2013; Meyers et al., 2014; Bird et al., 2019), apparently catalyzed by diverse microbes (Orsi et al., 2018). Notwithstanding the diversity of subsurface environments in which active extracellular enzymes have been observed, only a handful studies have addressed extracellular enzymes below ~20cm below the seafloor (Arnosti et al., 2014). The range, diversity, and controls on extracellular enzymes as a pathway for heterotrophic microbes to obtain organic carbon in subsurface environments remain largely unknown.

Here, we report the potential of extracellular enzymes to provide bioavailable organic carbon to the heterotrophic microbial communities down to 77.9mbsf in the International Ocean Discovery Program (IODP) Expedition 347, hole M0059C, in Baltic Sea sediment. We assayed potential extracellular enzyme activity using small substrate proxies for six different peptidases (enzymes that hydrolyze peptide bonds, e.g., in proteins), five different glycosyl hydrolases (enzymes that hydrolyze glycosidic bonds, as between sugars in a polysaccharide), and alkaline phosphatase, which cleaves phosphomonoesters, such as the phosphate head group on phospholipids. Hole M0059C spanned a glacial cycle of up to 44,000years (Andrén et al., 2015), with modern marine deposits underlain by lacustrine deposits below 50mbsf from the last glacial maximum. Total organic carbon was higher in the upper marine deposits (3–8%), than in the deeper lacustrine deposits (~1%), meaning that extracellular enzymes would have very different substrate pools above and below 50mbsf. We found that the extracellular enzyme activity profiles were different in these different substrate pools, and enzymes were highly stable, retaining their activity after autoclaving.

## Materials and Methods

### Site Description and Sample Collection

Sediments were collected from hole M0059C as part of IODP Expedition 347 (Andrén et al., 2015) on September 19, 2013. Site M0059 sits in the southern Little Belt, an incised valley between the island of Als, off the Danish Jutland peninsula, and the island of Fyn. Hole M0059C is at 55°0.29′N, 10°6.49′E under 37.1m of brackish water. The sediments analyzed in this study consist of brackish/marine sediments deposited in the past ~7,500years, overlaying freshwater glacial lake sediments deposited since the beginning of the Holocene. Samples were collected *via* piston coring system and stored at −80°C from the time of collection until analysis. Depths from which samples were selected and corresponding stratigraphic units and sediment ages as determined by Van Helmond et al. (2017) are listed in Table 1.

**Table 1:** Depths sampled for enzyme activities, corresponding ages interpolated from the model of van Helmond et al. (2017), and stratigraphic units as described by Andrén et al. (2015).

Unit	Depth, mbsf	Age, yr. (max, min)
Ia	4.50	792 (656, 928)
	11.10	1784 (1,636, 1935)
	17.60	3,179 (3,000, 3,357)
	24.30	4,311 (4,125, 4,498)
	30.90	5,248 (5,052, 5,444)
	37.50	6,208 (6,064, 6,352)
	43.15	6,958 (6,807, 7,109)
Ib	48.22	7,401 (7,273, 7,528)
Unit II: no samples taken; unconformity at 51.68–51.73mbsf		
III	54.95	n.a.
	61.55	n.a.
	77.92	n.a.

### Extracellular Enzyme Assays

Extracellular enzymes were assayed using small fluorogenic substrate proxies (Hoppe, 1983). Eleven different fluorogenic substrates were used to assay the activity of corresponding enzymes (Table 2): six substrates for peptidases, five substrates for glycosylases, and one substrate for alkaline phosphatase. Briefly, on the day of analysis, a 1-cm subsample was collected from frozen core rounds using an electric drill with an ethanol-sterilized hole saw bit. 3g of this subcore was immediately placed in a sterile serum vial under N_2_ to serve as a sterile control and autoclaved for 60min on a liquid cycle.

**Table 2:** Enzymes used in this study and the corresponding fluorogenic substrates.

Enzyme	Description	Substrate	E.C.
Leucyl aminopeptidase	Exopeptidase	L-Leucine-AMC	3.4.11.1
Arginyl aminopeptidase	Exopeptidase	L-arginine-AMC	3.4.11.6
Prolyl aminopeptidase	Exopeptidase	L-Proline-AMC	3.4.11.5
Ornithyl aminopeptidase	Exopeptidase	Ornithine-AMC	na^*^
Gingipain	Endopeptidase	Z-Phe-Arg-AMC	3.4.22.37
Clostripain	Endopeptidase	Z-Phe-Val-Arg-AMC	3.4.22.8
β-d-xylosidase	Glycosyl hydrolase	MUB-β-d-xylopyranoside	3.2.1.37
β-d-cellobiohydrolase	Glycosyl hydrolase	MUB-β-d-cellobioside	3.2.1.91
N-acetyl-β-d-glucosaminidase	Glycosyl hydrolase	MUB-N-acetyl-β-d-glucosaminide	3.2.1.52
β-glucosidase	Glycosyl hydrolase	MUB-β-d-glucopyranoside	3.2.1.21
α-glucosidase	Glycosyl hydrolase	MUB-α-d-glucopyranoside	3.2.1.20
Alkaline phosphatase	Phosphatase	MUB-PO_4_	3.1.3.1

* *We are not aware of a formally described enzyme whose primary function is to catalyze the hydrolysis of N-terminal ornithine from a broad set of proteins*.

*AMC stands for 7-amino-4-methylcoumarin. MUB stands for 4-methylumbelliferone. All amino acids are l stereoisomers*.

A separate 3g sample was thawed in 100ml of sterile, anoxic, and room-temperature 20mm borate-buffered saline (BBS) at pH 8.0. BBS was selected because it buffers reasonably well at pH 8.0 and does not competitively inhibit any of the enzymes included in this study, unlike phosphate buffer, which inhibits alkaline phosphatase, or many organic buffers, which contain amine groups that competitively inhibit peptidases. The thin slurry of 3g sediment in 100ml buffer was chosen to minimize enzyme inhibition by humic substances and other organics and to maximize light transmission through the slurry, based on preliminary methodological experiments (Schmidt, 2016) and following Bell et al. (2013). The autoclaved sediment was prepared identically. Enzyme assays were performed in an anaerobic glove box under a 100% N_2_ atmosphere.

We performed two classes of enzyme assay: *V_max_* measurements and saturation curves. *V_max_* measurements, which were designed to indicate maximum potential enzyme activity at saturating substrate concentrations, were made at a single concentration of substrate, 400μm, with three live and three autoclaved replicates. For *V_max_* experiments, 20μl of a 20mm substrate stock was added to 980μl sample slurry. Saturation curves were designed primarily to indicate the binding coefficient of enzymes to the fluorogenic substrate and were made by comparing hydrolysis rates measured in triplicate to autoclaved controls measured in triplicate, at each of 10 or 11 substrate concentrations spaced evenly from 0 to 720 or 800μm. An appropriate quantity of substrate stock was added to 940μl sample slurry, and pure water (glycosyl hydrolase and phosphatase substrates) or DMSO (peptidase substrates) was added to a total of 1,000μl, so that the slurry medium was identical between substrate concentrations. Standard curves were created with 7-amino-4-methylcoumarin (AMC) dissolved in DMSO or 4-methylumbelliferone (MUB) dissolved in pure water, added to sample slurries as described for the saturation curve measurements.

Substrates were added to each cuvette. The time of substrate addition constituted time zero. Fluorescence was measured as soon as possible after substrate addition using a Promega GloMax Multi JR single-cuvette fluorescence reader set to UV mode. We have found this approach to be both more precise than the more-common plate reader method (Bell et al., 2013) and to reduce fluorescence drift which yields apparently precise but spurious measures of enzyme activity (Schmidt, 2016). Fluorescence values were measured 3–5 times over the course of ~24h, and enzyme activities were calculated as described in Steen et al. (2019). Raw data and R scripts used to calculate results are posted at.^1^

Enzyme assays were performed at 20°C. *In situ* core temperatures were not measured, but bottom water temperature data from Little Belt indicates an average sediment temperature of 8°C (Ni et al., 2020). Data were analyzed following previously described procedures (Lloyd et al., 2013; Steen et al., 2019).

### Cell Counts

Cell counts were taken from data reported in Buongiorno et al., 2017. Because cell counts were made close to, but not precisely at, the same depths as enzyme activity measurements, cell count data at enzyme measurement depths were interpolated using locally fitted polynomials above and below the unconformity.

## Results

### Absolute and Cell-Specific Potential Enzyme Activities

**Figure 1:**
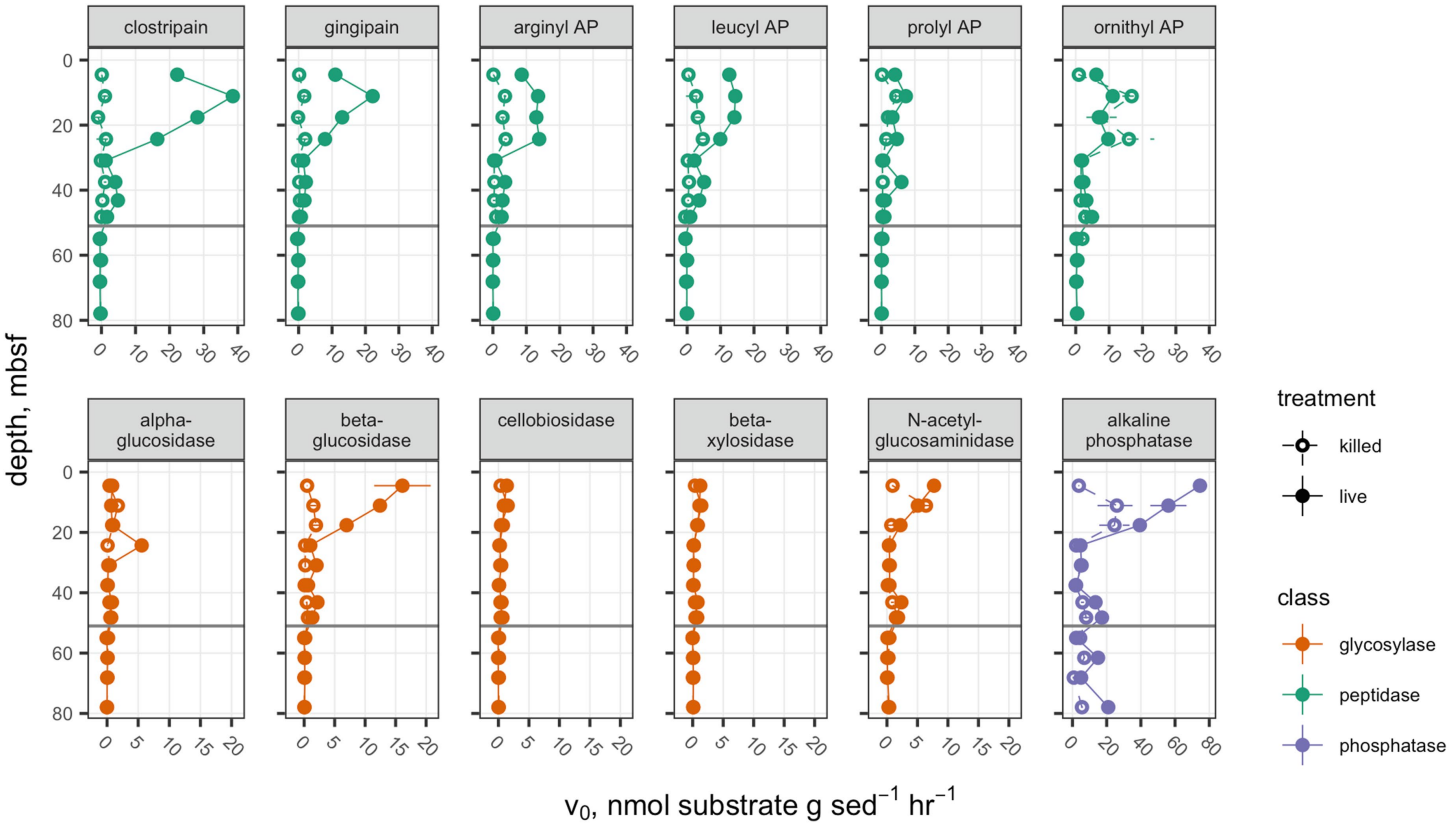
Potential enzyme activities in hole M0059C as a function of depth. Live sample *v_0_* data for clostripain, gingipain, arginyl AP, leucyl AP, prolyl AP, α-glucosidase, β-glucosidase, N-acetyl-β-d-glucosaminidase, and β-d-xylosidase were previously presented in Bird et al. (2018). Open circles indicate autoclaved killed controls; filled circles indicate live samples. Note that each enzyme class shares a common scale, but scales differ between enzyme classes. Error bars represent one standard deviation about the mean for triplicate measurements. In cases where the error bars are not visible, they are smaller than the marker.

Potential activities of each peptidase, with the exception of ornithyl aminopeptidase (Orn-AP), were greater than the autoclaved control above the unconformity at 51.7mbsf (Figure 1). Orn-AP was also elevated at depths above 51.7mbsf, but activities in “live” sediments were indistinguishable from those in autoclaved sediments. For each peptidase, potential activity increased from 4.3 to 11.1mbsf and then decreased gradually down to 30mbsf. Maximum potential activities in the live samples ranged from 22.2±0.1nmolg sed^−1^ h^−1^ for clostripain to 7.3±0.6nmolg sed^−1^ h^−1^ for prolyl aminopeptidase. Potential activities of peptidases also tended to increase downcore from a low value at 30.9mbsf to higher values between there and the unconformity at 51.7mbsf. Below that unconformity, in lacustrine sediments with very low organic carbon content buried during the last glacial maximum, potential activities were indistinguishable from zero.

Glycosyl hydrolases (polysaccharide-hydrolyzing enzymes) had lower maximum potential enzymatic rates and followed a different pattern with depth than peptidases. Only β-glucosidase showed a significant difference between the live and autoclaved samples at multiple depths. α-glucosidase potential activity was slightly greater than the autoclaved control at only 24.3mbsf, and N-acetyl-glucosaminidase was significantly greater than the killed control at only 4.3mbsf. Glycosyl hydrolases potential activities were, in general, considerably less active than aminopeptidases: The highest potential activity for any glycosylase was β-glucosidase at 4.5mbsf, with 16.1±4.6nmolg sed^−1^ h^−1^. They also did not increase in activity with depth in any part of the borehole. Similarly, to the peptidases, no activity was seen for glycosylases in sediments under 51.7mbsf of lacustrine origin.

Alkaline phosphatase potential activities were higher throughout the core than either of the other classes of enzymes, ranging from 74.6±3.2nmolg sed^−1^ h^−1^ at 4.5mbsf to 2.12±0.09nmolg sed^−1^ h^−1^, and this was the only enzyme for which potential activities were higher in the live sample than the killed control below the unconformity at 51.68mbsf.

**Figure 2:**
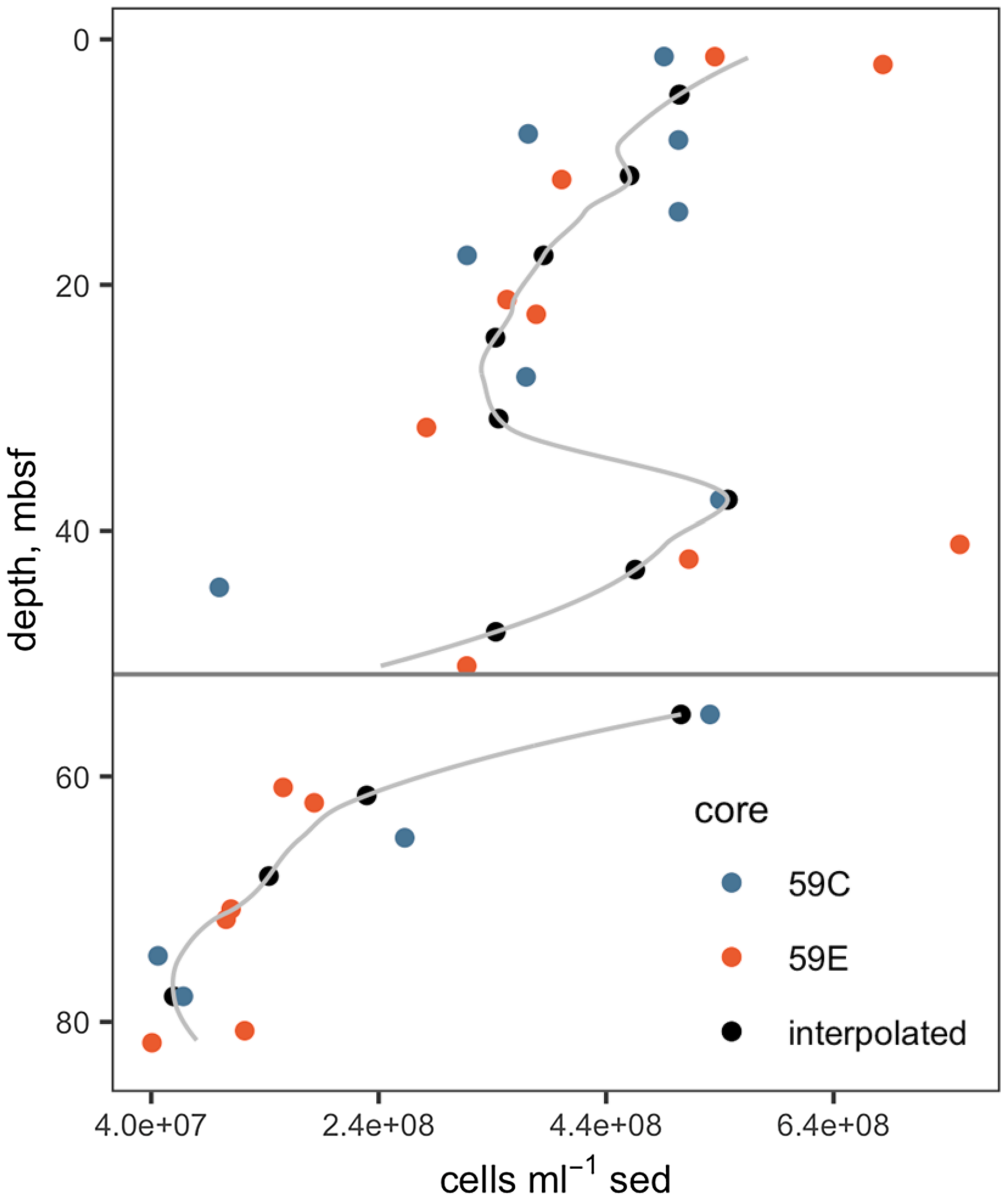
Cell abundances. Data from cores 59C and 59E were used in order to improve the precision of the interpolation. The gray line indicates the full interpolation, and the black points indicate the interpolated cell counts at depths at which enzyme activities were measured. The horizontal gray line indicates the unconformity. Underlying cell count data are from Buongiorno et al., (2017).

**Figure 3:**
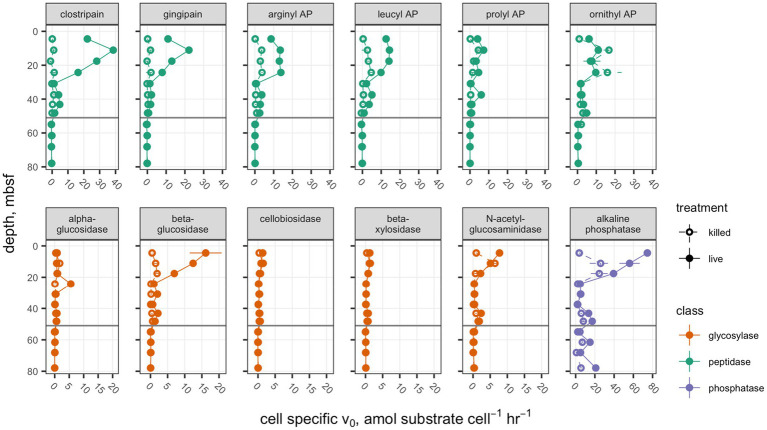
Cell-specific potential enzyme activities. Presentation is the same as Figure 1.

Interpolated cell abundance values decreased from 4.6×10^8^ cells g^−1^ sed at 4.35mbsf, to 6.0×10^7^ cells g^−1^ at 78mbsf (Figure 2), with the result that patterns of cell-specific potential enzyme activities were qualitatively similar to absolute potential activities (Figure 3). Cell-specific activities ranged from a maximum of 151±3.5 fmol cell^−1^ g sed^−1^ h^−1^ for clostripain at 11.1mbsf to undetectable for most enzymes below the unconformity.

### Saturation Curves

Saturation curves were measured for each enzyme in the shallowest sample, 4.5mbsf. *K_m_* values could only be calculated for a subset of enzymes (Table 3); others exhibited activity that was either indistinguishable from zero or low and not qualitatively consistent with Michaelis-Menten kinetics.

**Table 3:** *K*_m_ values for enzymes at 4.5mbsf.

Enzyme	*K_m_*, μm (±std. err)
Clostripain	200±37.5
Gingipain	69.5±38.1
Arginyl AP	61.1±27.3
Leucyl AP	73.0±53.3
Prolyl AP	168±30.4
Ornithyl AP	997±489
α-glucosidase	n.d.
β-glucosidase	230±72.5
Cellobiosidase	n.d.
b-xylosidase	n.d.
N-acetylglucosidase	351±128
Alkaline phosphatase	1,013±613

*Error values given are standard error of the parameter estimate*.

**Figure 4:**
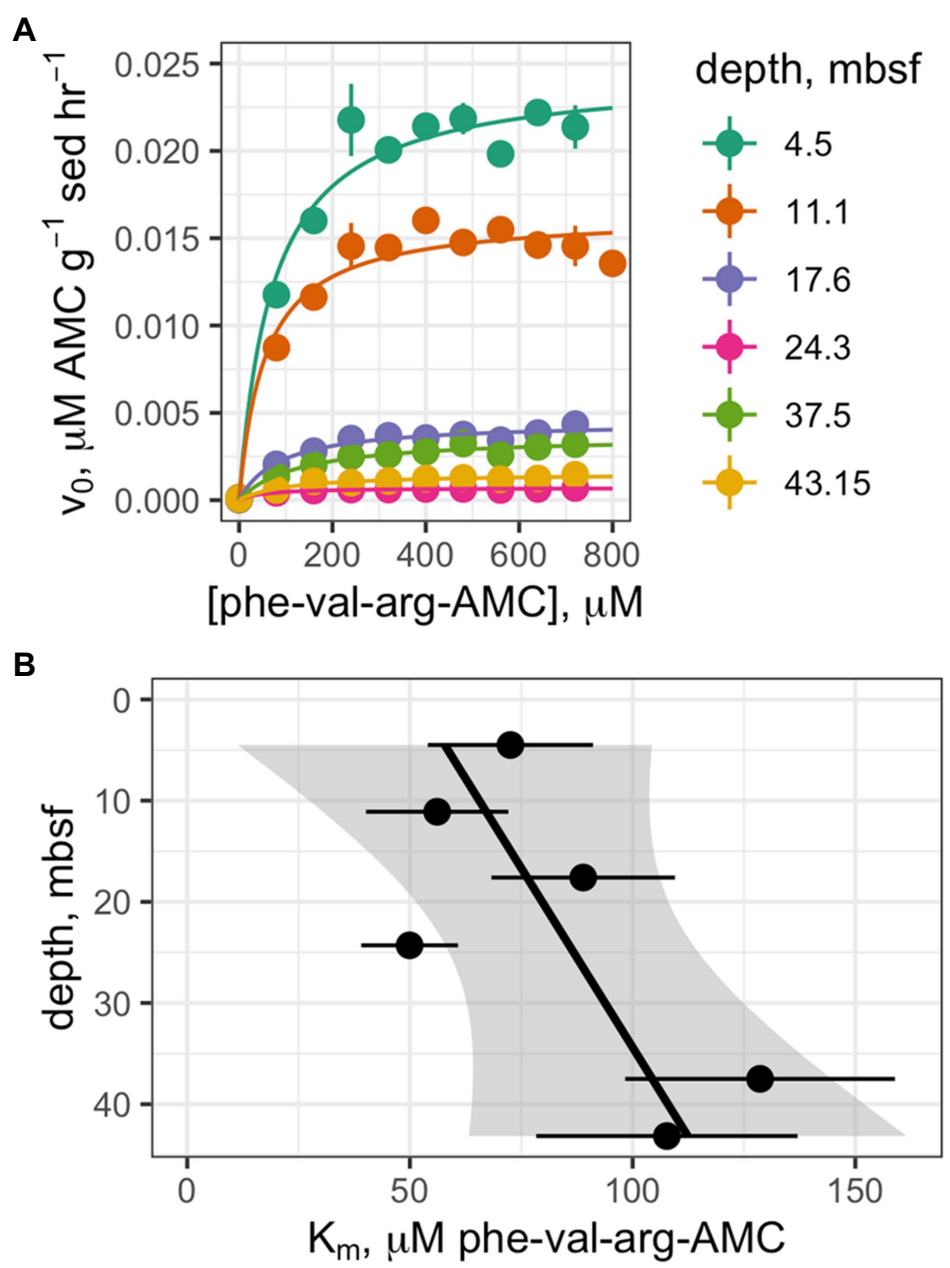
**(A)** Saturation curves of Z-phe-val-arg-AMC (clostripain substrate) at six depths. **(B)** Trend of *K_m_* values as a function of depth.

Saturation curves were measured for one enzyme, clostripain, at six depths which were selected on the basis of having clearly detectable enzyme activity (Figure 4A). The hydrolysis rate data qualitatively fit well to a Michaelis-Menten function at all depths. Despite the fact that *V_max_* values decreased by 94% with depth, calculated apparent *K_m_* values did not change significantly with depth (Figure 4B; *p* =0.12, *n* =6).

### Activity in Autoclaved Controls

**Figure 5:**
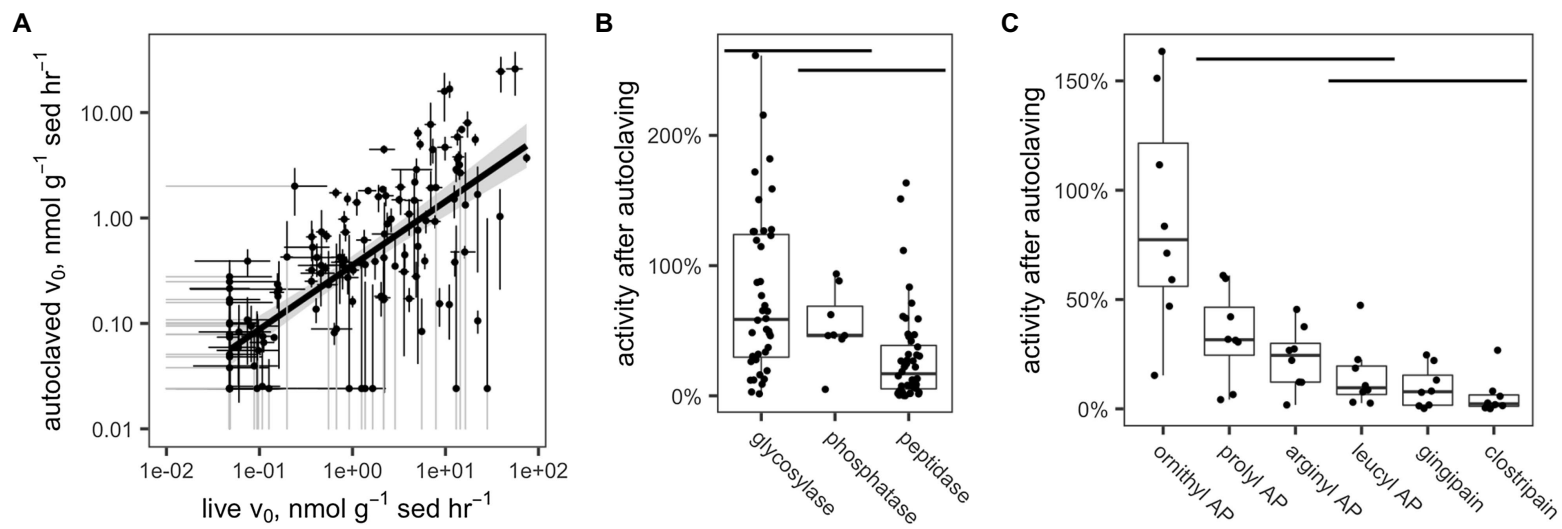
**(A)** Measured hydrolysis rates of live vs. autoclaved sediments. In order to increase legibility on the log-log plot, v_0_ values have been adjusted to the detection limit of 4.77×10^−2^n molg sed^−1^ h^−1^ (live sediments) or 2.41×10^−2^n molg sed^−1^ h^−1^ (killed sediments). Standard error bars that dip below 1×10^−2^μ molg sed^−1^ h^−1^ are truncated and colored gray. **(B,C)** Fraction of enzyme activity remaining after autoclaving by **(B)** enzyme class and **(C)** enzyme, within the peptidase class. Horizontal bars indicate distributions that are statistically indistinguishable (*α*=0.05, Conover-Iman test).

Potential enzyme activities in live samples were correlated with activities in the corresponding autoclaved samples (Figure 5A). For all enzymes and samples (excluding those for which activity was close to the detection limit), a median of 51.3% of activity was retained after autoclaving (interquartile range: 11.7–66.4%). These results differed significantly by enzyme class (Figure 5B; *p* <0.001, Kruskal-Wallis test, *n* =96): Glycosylases retained a median of 77.2% (IQR 29.7–124%), phosphatase retained a median of 54.0% (IQR 45.6–68.8%), and peptidases retained a median of 29.2% (5.38–38.7%). Only the differences between glycosylases and peptidases and between phosphatase and peptidases were statistically significant (*p* <0.001 and *p* <0.01, respectively, Conover-Iman test using the Conover test package in R; Dinno, 2017).

Within the peptidases, there were also significant differences among enzymes in terms of activity retained after autoclaving (Figure 5C). Ornithyl AP retained the most activity (median=87.8%, IQR 56–122%), while leucyl AP, clostripain, and gingipain, the distributions of which did not differ significantly, retained the least (leucyl AP: 15.1% [IQR: 6.58–19.6%], gingipain: 9.87% [IQR: 1.67–15.4%], and clostripain: 5.91% [IQR: 1.22–6.35%]).

## Discussion

Activities of a broad range of extracellular enzymes were detectable to a depth of 48mbsf. The presence of active extracellular enzymes at these depths agree with previous work showing the presence of mRNA transcripts of extracellular hydrolases in similarly deep subsurface marine sediments (Zinke et al., 2017, 2019; Orsi et al., 2018; Bird et al., 2019). It is challenging to know precisely what range of substrates is present in these sediments, since it is difficult to precisely characterize the large diversity of organic molecules buried in marine sediments (Hedges et al., 2000; Wakeham and Lee, 2019). It is also unknown what range of substrates can be hydrolyzed by each of the enzymes assayed here, since many extracellular enzymes have been shown to hydrolyze a wide range of substrates (Steen et al., 2015). However, the general classes of substrates that we analyze here – polysaccharides, proteins, and phospholipids – are ubiquitously present in sedimentary organic matter and a fraction of them are enzymatically labile (Dauwe et al., 1999; LaRowe et al., 2020). Thus, these enzymes are active in the presence of environmentally relevant substrates and therefore must produce a flux of low molecular weight organic carbon to the *in situ* heterotrophic microbial community.

Previous work in these sediments has shown that hydrolysis of complex organic matter appears to be central to the life strategy of the *Atribacteria*, one of the abundant microbes in these sediments. Furthermore, Atribacteria may exist in commensal relationships with other species by “sharing” amino acids, perhaps in return for some other resource (Bird et al., 2019). Our data suggest that at least six types of peptidases are more active than glycosyl hydrolases. This may indicate that these communities are primed to access complex extracellular proteins over polysaccharides because peptides bring nitrogen along with the carbon into the cellular metabolism during catalysis. It is also possible that glycosyl hydrolases that do not act on the five different small fluorogenic substrate proxies used here were present. The activity of multiple, extracellular hydrolytic enzymes as well as the molecular evidence for metabolism of the products by diverse members of the subsurface community highlight the importance of complex organic carbon metabolism to subsurface ecosystems.

Enzyme assays were performed 12°C above *in situ* temperatures for logistical reasons and to maximize the chance of measuring any enzymes that might have been present. Reports of the temperature response of marine enzymes vary widely, but a reasonable approximation is that enzyme activities tend to increase by a factor of 2 when temperature increases by 10°C. That would imply that the *V_max_* values measured here are higher than *in situ* values by a factor of 2.4. It is likely that the different enzymes measured here are affected differently by the increase in temperature, but the differences among enzyme classes are not likely large enough to be responsible for, for instance, the factor-of-four difference between the most active peptidase (clostripain) and the most active glycosylase (β-glucosidase).

The absence of detectable activity of extracellular enzymes for complex organic matter other than phosphomonoesters below the unconformity in lacustrine sediments of the last glacial maximum is consistent with much lower concentrations of organic matter at these depths (Andrén et al., 2015; Marshall et al., 2018) and lower transcript abundances of genes for organic matter hydrolysis (Zinke et al., 2019). This result, coupled to a higher percentage of genes from the Wood-Ljungdahl carbon fixation pathway in the lacustrine sediments (Marshall et al., 2018), suggests that perhaps autotrophy is more important for the microbial communities at these depths. This would be consistent with the evidence of phosphatase activity below the unconformity at 51.7mbsf, where autotrophs would still require phosphorus.

We note that absence of evidence of glycosylases below the unconformity is not necessarily evidence of absence, however. Polysaccharides and glycosyl hydrolases are both highly diverse, and it is possible that there were glycosyl hydrolases present in sediments that did not hydrolyze the fluorogenic substrate proxies used here.

Broadly, peptidase activities increased from 4.5mbsf to 11.1mbsf and then decreased to the unconformity. 4mbsf is already well within the “deep subsurface” because these 4.5-m deep sediments are approximately 790years old (Kotthoff et al., 2017), do not have regular replenishment of oxidants and fresh organic matter, and experience a steady decline in total cellular abundance with depth (Figure 2). Multiple scenarios could account for this increase in peptidase activities with depth within the deep subsurface. It is possible that the microbial communities at 11.1mbsf simply produce more enzymes than those at 4mbsf, although why that would be is a mystery: TOC decreased from 7.54% at 4.38mbsf to 5.79% at 11.27mbsf (Egger et al., 2017), and carbon oxidation rates tend to decrease with decreasing TOC and increasing depth (Beulig et al., 2018; Jørgensen et al., 2020).

Another factor that could contribute to this rise in enzyme activity with depth could be the release of cytoplasmic peptidases during cell rupture associated with cell depth between 4mbsf and 11mbsf. Such “living dead” enzymes (i.e., active enzymes released from dead cells) have been hypothesized to provide labile, low molecular weight carbon to deep-sea communities (Baltar, 2018; Baltar et al., 2019). A similar dynamic in the subsurface would act as a negative feedback on cell death by starvation in subsurface sediments, in that cells dying due to lack of carbon substrates would release enzymes that would provide such substrates to the surviving community members. The amount of organic carbon in these dying cells is well below what would be required to support the community, suggesting that buried organic matter must also serve as substrates (Bradley et al., 2018), but the amount of labile carbon that necromass-derived enzymes could liberate from sedimentary organic matter is as-of-yet unconstrained. There may be a second increase in enzyme activity with depth between 30 and 50mbsf that might be driven by similar mechanisms, but this is small and could be noise.

Given the sediment accumulation rate of ~60cm per 100years (van Helmond et al., 2017), accumulation of extracellular enzymes over macroscopic depth scales could only happen if those enzymes are stable on the timescales of tens to thousands of years. While this might appear to be an extremely long time for enzymes to remain active, in the next section we show that these extracellular enzymes may indeed be extraordinarily stable.

### Long Lifetimes of Extracellular Enzymes in Subsurface Sediments

Two lines of evidence indicate that, once released by active secretion or cellular death, active extracellular enzymes must persist in sediments over long timescales. First, the persistence of enzyme activity after autoclaving indicates that enzymes are very stable. Soil enzymes have been observed to retain some activity after autoclaving (Carter et al., 2007; but see Blankinship et al., 2014 for a counterexample). This is assumed to be because a relatively large number of non-covalent interactions between enzymes and clay mineral surfaces (Arnarson and Keil, 2000; Johnston et al., 2012) prevent denaturation of enzymes at high temperature. It is also possible that these enzymes do denature upon autoclaving but re-fold into an active conformation when they cool. That would suggest that denatured enzymes are likely to re-enter a correctly folded state, which again would point toward stability under *in situ* conditions. A third possibility is that the substrate hydrolysis observed in these experiments was primarily catalyzed by abiotic factors, e.g., manganese oxide (Reardon et al., 2016). However, an abiotic mechanism would not be expected to follow Michaelis-Menten kinetics, whereas the hydrolysis rates observed here generally did. Another possibility would be that intact cells, rather than just their enzymes survive autoclaving. Autoclaving kills most of the cells in a natural sample (Tuominen et al., 1994; Otte et al., 2018); however, some types of spores (O’Sullivan et al., 2015) have been shown to maintain a fraction of their activity after autoclaving.

A second, theoretical line of evidence also indicates that extracellular enzyme lifetimes must be unusually long when microbial metabolisms are slow, as in these sediments. In order for enzyme production to represent a viable strategy to obtain resources, enzymes must “pay for themselves” in terms of the relevant resource (Vetter et al., 1998). This puts a lower limit on the amount of hydrolysate that an enzyme must produce over its lifetime: If we assume that carbon is the relevant resource an enzyme produces, then the enzyme must return as much carbon to the community as the enzyme itself contains. An upper limit on the amount of hydrolysate an enzyme produces over its lifetime comes from the fact that hydrolysis products, such as free amino acids, and sugars do not generally accumulate in sediments (e.g., Dauwe and Middelburg, 1998). Exometabolomics measurements on M0059 identified no free amino acids, suggesting that they are not accumulating in these sediments (Bird et al., 2019). Thus, the total rate of hydrolysate production by enzymes cannot exceed the community metabolic rate, including respired C and biomass production.

Let us imagine that enzyme production and degradation are in quasi-steady state. If we imagine that all biomass production goes toward producing enzymes, then biomass production equals enzyme degradation rate, so we can constrain the turnover time of enzymes, τ, as:

$$\tau \geq \frac{\mathrm{enzyme concentration}}{\mathrm{community biomass production rate}}$$ (1)

where enzyme concentration and community biomass production rate are both expressed in C units. Community respiration rates (e.g., sulfate reduction rates) are more easily measured than biomass production rates, so this can also be expressed in terms of community respiration and growth efficiency, *GE*. When GE is low, as in the subsurface, GE = biomass production rate/respiration rate, so that:

$$\tau \geq \frac{\mathrm{enzyme concentration}}{\mathrm{GE}\times \mathrm{community respiration rate}}$$ (2)

Thus, for a fixed enzyme concentration, enzyme turnover times – and thus enzyme lifetimes – scale inversely with community respiration rates. It is extremely challenging to precisely measure either enzyme concentration or growth efficiency in the subsurface, but growth efficiency can be assumed to be low (Hoehler and Jørgensen, 2013; Jørgensen and Marshall, 2016). Enzyme concentrations in sediments can be constrained using specific activities (*V_max_* per mol of enzyme), which are sometimes reported for purified enzymes in buffer solutions:

$$\begin{array}{l} \mathrm{enzyme concentration}\;\left(\mathrm{mol}\;C\;g\;{\mathrm{sed}}^{-1}\right)\\ =\frac{\mathrm{potential activity}\;\left(\mathrm{mol}\;\mathrm{bonds}\;{\mathrm{hr}}^{-1}\;g\;{\mathrm{sed}}^{-1}\right)}{\mathrm{specific activity}\;\left(\mathrm{mol}\;\mathrm{bonds}\;{\mathrm{hr}}^{-1}\;\mathrm{mol}\;C\;{\mathrm{enzyme}}^{-1}\right)}. \end{array}$$ (3)

Thus, enzyme half-life is bounded by:

$$\tau \;\left(\mathrm{hr}\right)\geq \frac{V_{\max}\;\left(\mathrm{mol}\;\mathrm{bonds}\;{\mathrm{hr}}^{-1}\;g\;{\mathrm{sed}}^{-1}\right)}{\begin{array}{l} \mathrm{[specific activity}\;{\left(\mathrm{mol}\;\mathrm{bonds}\;\mathrm{hr}\right)}^{-1}\;{\left(\mathrm{mol}\;C\;\mathrm{enzyme}\right)}^{-1}\times \\ \mathrm{GE}\times \mathrm{community respiration rate}\;\left(\mathrm{mol}\;C\;{\mathrm{hr}}^{-1}g\;{\mathrm{sed}}^{-1}\right)] \end{array}}$$ (4)

This formula will almost certainly yield an underestimate of enzyme concentration, because specific activities of enzymes in sediments will be reduced due to interactions with mineral surfaces and humic substances compared to activities in buffer solution (Boavida and Wetzel, 1998; Tietjen and Wetzel, 2003). This effect would not be relevant to the *V_max_* term because our measurements of *V_max_* would reflect any suppression of enzyme activity by mineral sorption.

Using a range of reported enzyme-specific activities and assuming that community carbon oxidation rates are on the order of 0.1nmolcm^−3^ day^−1^ (Beulig et al., 2018), that bacterial growth efficiency is 10% (likely an overestimate) and that surface/humic interactions have no effect, we calculate a median clostripain lifetime of 230days (see supplemental for a more detailed description of these calculations). Considering that both the growth efficiency is likely an overestimate and that 100% of biomass production is not invested in clostripain alone, this value represents a lower-bound estimate for clostripain lifetime. For instance, a conservative guess is that sorption to surfaces reduces specific activity by a factor of three, and del Giorgio and Cole estimate a minimum growth efficiency of 3.7% in seawater. Accounting for these two factors would increase estimated minimum enzyme lifetimes by a factor of 10. Just as microbial metabolic rates in the subsurface are orders of magnitude slower than what can be observed in pure cultures, it seems that enzyme lifetimes in the subsurface are substantially longer than what we typically observe for purified enzymes.

The active respiration of electron acceptors, such as oxygen, nitrate, iron, sulfate, and carbon dioxide in deep subsurface marine sediments, has been well-established (D’Hondt et al., 2004). However, direct measurements of the activity of the organic matter presumed to donate the electrons for these respiratory metabolisms are less well-studied. We conclude that the microbial community in sediments up to 50mbsf in the Baltic Sea Basin metabolizes complex, old, and diverse organic matter. This organic matter is complex because extracellular enzymes are used to degrade it. It is old because the sediments are old and no advective processes introduce fresh organic matter into the system. It is diverse because a range of substrates are metabolized by these active extracellular enzymes. Furthermore, the enzymes that perform these functions are very stable. Stability of purified enzymes is usually measured with lifetimes of hours or days. Our results suggest that extracellular enzymes in the Baltic Sea deep subsurface sediments may be stable for months or years, and possibly much longer.

## Data Availability Statement

The raw data supporting the conclusions of this article and R scripts used to process those data are available at https://github.com/adsteen/IODP_347_enzymes.

## Author Contributions

AS designed the experiments, analyzed the data, and wrote the manuscript. JS performed the analyses. TR estimated lifetimes of extracellular enzymes. KL analyzed the data and wrote the manuscript. All authors contributed to the article and approved the submitted version.

## Funding

Funding for this project was provided by the NSF (OCE-1431498), the NSF Center for Dark energy Biosphere Investigations (OCE-0939564 contribution #576), and Department of Energy, Office of Science, Office of Biological and Environmental Research (DE-SC0020369) to AS and KL.

## Conflict of Interest

The authors declare that the research was conducted in the absence of any commercial or financial relationships that could be construed as a potential conflict of interest.

## Publisher’s Note

All claims expressed in this article are solely those of the authors and do not necessarily represent those of their affiliated organizations, or those of the publisher, the editors and the reviewers. Any product that may be evaluated in this article, or claim that may be made by its manufacturer, is not guaranteed or endorsed by the publisher.

## Footnotes

^1^ https://github.com/adsteen/IODP_347_enzymes
