# Supplementary material for: Potential Activities and Long Lifetimes of Organic Carbon-Degrading Extracellular Enzymes in Deep Subsurface Sediments of the Baltic Sea
Source: Front Microbiol. 2021 Sep 17;12:702015. doi: 10.3389/fmicb.2021.702015 (PMC8485070; doi:10.3389/fmicb.2021.702015)
Supplement: Supplementary file 1 [file Data_Sheet_1.PDF]

Constraining the lifetime of extracellular enzymes in subsurface sediments.

Jenna M. Schmidt Taylor M. Royalty Karen G. Lloyd

Andrew D. Steen
asteen1@utk.edu

A steady-state model of extracellular enzyme lifetimes

As described in the main text, we can imagine extracellular enzyme production and degradation to be in steady state. This allows us to imagine the situation as a reservoir turnover time calculation, where we make the approximation that enzyme lifetime is equivalent to the turnovertime of enzymes in the reservoir. Thus, the turnover time τ of enzymes must be:

$$\tau = \frac{\text{enzyme concentration}}{\text{enzyme production rate}} \tag{1}$$

Assuming that 100% of biomass production goes into the production of a *single* extracellular enzyme, the the lower limit of enzyme lifetime is given by

$$\tau = \frac{\text{enzyme concentration}}{\text{biomass production rate}} \tag{2}$$

Growth efficiency, GE, is defined as

$$\text{GE} = \frac{\text{biomass production rate}}{\text{biomass production rate} + \text{respiration rate}} \tag{3}$$

When biomass production is low relative to respiration, this simplifies to

$$\text{GE} = \frac{\text{biomass production rate}}{\text{respiration rate}} \tag{4}$$

Combining equation 2 with equation 4, the lower limit of enzyme lifetime is

$$\tau = \frac{\text{enzyme concentration}}{\text{GE} \times \text{respiration rate}} \tag{5}$$

Here, enzyme concentration is in units of mol C per unit volume (or mass) of sediment and community respiration is in units of mol C per unit volume (or mass) of sediment per unit time.

Parameter	Units
V_{max}	mol bonds hr^{-1} (g sed)$^{-1}$
specific activity	mol bonds (hr)$^{-1}$(mol C in enzyme)$^{-1}$
Growth efficiency (GE)	dimensionless
community respiration rate	mol C (hr)$^{-1}$(g sed)$^{-1}$

Table 1: Units required to calculate enzyme lifetime τ using equation 7.

Calculating enzyme concentration from $\mathbf{V_{max}}$ and specific activity

The concentration of extracellular enzymes cannot easily be measured, but it can be estimated from potential activity (V_{max}) and specific activity, which is V_{max} per unit enzyme by mass:

$$\text{enzyme concentration (mol C g}^{-1}\text{ sed}^{-1}) = \frac{V_{max}\text{ (mol bonds hr}^{-1}\text{g sed}^{-1})}{\text{specific activity (mol bonds hr}^{-1}\text{mol C enzyme}^{-1})} \tag{6}$$

Thus, the final equation for minimum enzyme lifetime τ is:

$$\tau = \frac{V_{max}}{\text{specific activity} \times \text{GE} \times \text{community respiration rate}} \tag{7}$$

with units given in table 1.

Estimating model parameters

None of these parameters can really be measured directly. In the back-of-the-envelope calculation described below, we will choose parameters conservatively, to systematically bias the result towards shorter lifetimes times and thereby calculate a lower bound on true enzyme lifetime.

$\mathbf{V_{max}}$

We have described measuring potential activity, or V_{max}, in the main text. There are two important technical points to keep in mind. First, we imagine the measured V_{max} to reflect a single enzyme. In fact, it measures not just a set of isoenzymes (i.e., structurally distinct enzymes that catalyze the same reaction) but also promiscuous enzymes capable of hydrolyzing the fluorogenic substrate as non-target substrates [Steen et al., 2015, Baltar, 2018].
Second, our model imagines only one enzyme in the system. In reality, subsurface communities produce many extracellular enzymes, in order to access organic C and other resources from a diverse set of macromolecules in subsurface OM. A more realistic model would account for the fact that each enzyme is satisfying only a fraction of the community resource demand. Our model

imagines that a single enzyme satisfies the community's entire organic carbon demand. In reality, each enzyme satisfies only a fraction of that demand, which implies a longer lifetime in order to justify its smaller 'return on investment'. In order to keep the model simple and to bias it towards shorter lifetimes, here we only consider a single enzyme.

Specific activity

As described above, we can estimate enzyme concentration from potential (i.e., V_{max}. To do this, we must assume a specific activity of the enzyme, i.e., the enzyme activity per mass of enzyme, as well as a carbon content of the enzyme. We can use literature values of specific activity measured for comparable enzymes. For this example, we will use the example of a cold-active bacterial trypsin isolated by Wang et al [Wang et al., 2005].
We will assume that the enzymes described in the paper here have similar specific activities. However, we will have to make two corrections:

- We will have to calculate what the specific activity would have been at the temperature at which we measured V_{max} *in situ*.
- We will have to convert the units in which specific activity is give - in this case, mass of product released per hour per mass of enzyme - into units appropriate for our calculation, namely (nano)moles of bonds hjydrolyzed per hour per mol C in enzymes per gram sediment.

Correcting specific activity for temperature

Wang et al characterized a cold-active bacterial trypsin that had a specific activity of 13,826 units of activity per mg enzyme at 35 °C, and 30 % of that at 0 °C [Wang et al., 2005]. They define a unit of activity as the quantity of enzyme required to liberate one µg tyrosine per minute.
The relationship between temperature and enzyme activity typically follows the Arrhenius relationship below a certain limit, which is often expressed in terms of Q_{10}, the factor by which enzyme activity increases upon a temperature increase of 10 °C:

$$Q_{10} = \left(\frac{r_2}{r_1}\right)^{\frac{10}{T_2 - T_1}} \tag{8}$$

Given that the measured specific activity at 0 °C was 30% of that at 35 °C, we can calculate Q_{10}, and then use that value to calculate the activity at 20 °C:

$$\begin{aligned} Q_{10} &= \left(\frac{1}{0.3}\right)^{\frac{10}{35}} \\ Q_{10} &= 1.410 \end{aligned} \tag{9}$$

Now that we know Q_{10}, we can rearrange Equation 8 to calculate the specific activity at 20 °C:

$$
\begin{aligned}
r_{20} &= 10^{\left(\log r_{35} - \frac{(T_{35}-T_{20})\log Q_{10}}{10}\right)} \\
&= 10^{(\log 13826 - 1.5\log 1.41)} \\
\mathbf{r_{20}} &= \mathbf{8,258\ U}
\end{aligned}
\tag{10}
$$

Converting specific activity units

The remaining task is then to convert specific activity from the units in which it was reported, µg tyrosine per hour per mg of enzyme, to the units that make sense here, (nano)moles of C in substrate released per hour per (nano)mole C of enzyme.

We can do this unit conversion for the enzyme described by Wang et al (2005) as follows[1]. Note that we use the rule of thumb that enzymes are 50% C by mass. The C content of amino acids by mass ranges from 36% (Ser and Ala) to 65% (Phe and Trp), with an average of 46%, so 50% is a reasonable approximation. The amino acid tyrosine is abbreviated as Y:

$$
\begin{aligned}
&\frac{8,258\text{ U enzyme}}{\text{mg enzyme}} \times \frac{1\mu\text{g Y min}^{-1}}{1\text{ U enzyme}} \times \frac{60\text{min}}{1\text{hr}} \times \frac{1\ \mu\text{mol Y}}{181\ \mu\text{g Y}} \\
&\times \frac{1000\text{ nmol Y}}{1\ \mu\text{mol Y}} \times \frac{1\text{ nmol bonds}}{1\text{ nmol Y}} \times \frac{1000\text{ mg enzyme}}{1\text{ g enzyme}} \times \frac{2\text{ g enzyme}}{1\text{ g C in enzyme}} \\
&\times \frac{12\text{ g C in enzyme}}{1\text{ mol C in enzyme}} \times \frac{1\text{ mol C in enzyme}}{10^9\text{nmol C in enzyme}} \\
&= \frac{\mathbf{66\ nmol\ bond\ hr^{-1}}}{\mathbf{nmol\ C\ in\ enzyme}}
\end{aligned}
\tag{11}
$$

For this calculation, we'll use the largest V_{max} measured in this study, clostripain at 11.1 mbsf, of 40 nmol substrate g sed^{-1} hr^{-1}.

Community respiration rate

We take community respiration rates from Bornhom Basin, where they have been measured carefully. There, respiration rates below 1 meter below the seafloor (mbsf) were less than 0.1 nmol cm^{-3}day^{-1}. Bornholm Basin is deeper than Little Belt, so community respiration rates in surface sediments are expected to be lower than in Little Belt surface sediments. On the other hand, we are discussing enzymes that are more than tenfold deeper in Little Belt sediments than the respiration rate at 1 mbsf measured in Bornholm Basin, so this still seems like a conservative estimate. We must convert this volumetric

[1] In general, specific activities are reported in idiosyncratic units, so the unit conversion is slightly different for each enzyme we report in table 2.

respiration rate estimate to units per mass sediment by assuming a sediment density of 2.5 g cm^{-3}:

$$\frac{0.1 \text{ nmol C}}{\text{cm}^3 \cdot \text{day}} \times \frac{1 \text{ cm}^3 \text{ sed}}{2.5 \text{ g sed}} \times \frac{1 \text{ day}}{24 \text{ hr}} = 1.7 \times 10^{-3} \text{ nmol C g sed}^{-1} \text{ hr}^{-1}$$

Growth efficiency

Growth efficiency is totally unknown. We will assume GE = 10%. GE is very likely to be less than 10% for the following reasons:

- GE tends to be lower when growth rates are low [del Giorgio and Cole, 1998]
- GE tends to be less than 10% in low-productivity marine environments such as the Sargasso Sea, which nevertheless feature higher bacterial production than the subsurface sediments discussed here [del Giorgio and Cole, 1998]
- The communities disscussed here appear to be in a state of near stasis, and do not on net produce new biomass [Jørgensen and Marshall, 2016].

Thus, GE = 10 % seems like a conservative estimate.

Calculating enzyme lifetime

We plug all of these measured and assumed parameters into equation 7 as follows:

$$\begin{aligned}\tau &= \frac{V_{max}}{\text{specific activity} \times \text{GE} \times \text{community respiration rate}} \\ &= \frac{40 \text{ nmol bonds } (\text{hr})^{-1} \text{ (g sed)}^{-1}}{66 \text{ nmol bond } (\text{hr})^{-1} (\text{nmol C})^{-1} \times 0.1 \times 1.7 \times 10^{-3} \text{ nmol C (g sed)}^{-1} \text{ hr}^{-1}} \\ \tau &= \mathbf{3,600 \text{ hours}}\end{aligned} \tag{12}$$

As we note in the text, Del Giorgio and Cole created a model of GE as a function of primary productivity that implied a physiological minimum GE of 3.7%, which is probably closer to sedimentary values than the 10% value we used. It is also reasonable to guess that specific activity is lower by a factor of at least 3 in the sedimentary environment versus *in vitro*, although the magnitude and sometimes direction of the change in enzyme activity in upon sorption to minerals is highly variable [Tietjen and Wetzel, 2003]. Thus, this estimate could easily be an underestimate enzyme lifetimes by a factor of ten.

Estimating tau using different specific activities

We have compiled a small set of specific activities for bacterial peptidases that have been reported in the literature. Using equation 7 after appropriate unit conversions, we find the following estimates of enzyme lifetimes:

specific activity<br>mol bonds $(hr)^{-1}$(mol C in enzyme)$^{-1}$	τ<br>hr	τ<br>year	ref
66	$3,600$	0.410	[Wang et al., 2005]
19	$13,000$	1.400	[Wang et al., 2008]
230	$1,000$	0.120	[Szwajcer-Dey et al., 1992]
94	$2,500$	0.290	[Chevallier et al., 1992]
31	$7,600$	0.860	[Ullmann and Jakubke, 1994]
0.002	$100,000,000$	$12,000$	[McLuskey et al., 2016]

Table 2: Calculated values of τ for specific activities of a sample of peptidases reported in the literature. All values have been rounded to two significant digits.

dThe R code used to calculate these lifetimes is posted at `https://github.com/adsteen/IODP_347_enzymes`.

References

[Baltar, 2018] Baltar, F. (2018). Watch out for the "living dead": cell-free enzymes and their fate. *Frontiers in microbiology*, 8:2438.

[Chevallier et al., 1992] Chevallier, S., Goeltz, P., Thibault, P., Banville, D., and Gagnon, J. (1992). Characterization of a prolyl endopeptidase from Flavobacterium meningosepticum. Complete sequence and localization of the active-site serine. *Journal of Biological Chemistry*, 267(12):8192–8199.

[del Giorgio and Cole, 1998] del Giorgio, P. a. and Cole, J. J. (1998). Bacterial Growth Efficiency in Natural Aquatic Systems. *Annual Review of Ecology and Systematics*, 29(1):503–541.

[Jørgensen and Marshall, 2016] Jørgensen, B. B. and Marshall, I. P. G. (2016). Slow Microbial Life in the Seabed. *Annual review of marine science*, 8:311–32.

[McLuskey et al., 2016] McLuskey, K., Grewal, J. S., Das, D., Godzik, A., Lesley, S. A., Deacon, A. M., Coombs, G. H., Elsliger, M. A., Wilson, I. A., and Mottram, J. C. (2016). Crystal structure and activity studies of the C11 cysteine peptidase from parabacteroides merdae in the human gut microbiome. *Journal of Biological Chemistry*, 291(18):9482–9491.

[Steen et al., 2015] Steen, A. D., Vazin, J. P., Hagen, S. M., Mulligan, K. H., and Wilhelm, S. W. (2015). Substrate specificity of aquatic extracellular peptidases assessed by competitive inhibition assays using synthetic substrates. *Aquatic Microbial Ecology*, 75(3):271–281.

[Szwajcer-Dey et al., 1992] Szwajcer-Dey, E., Rasmussen, J., Meldal, M., and Breddam, K. (1992). Proline-specific endopeptidases from microbial sources: Isolation of an enzyme from a Xanthomonas sp. *Journal of Bacteriology*, 174(8):2454–2459.

[Tietjen and Wetzel, 2003] Tietjen, T. and Wetzel, R. G. (2003). Extracellular enzyme-clay mineral complexes: Enzyme adsorption, alteration of enzyme activity, and protection from photodegradation. *Aquatic Ecology*, 37(4):331–339.

[Ullmann and Jakubke, 1994] Ullmann, D. and Jakubke, H. D. (1994). Kinetic characterization of affinity chromatography purified clostripain. *Biological Chemistry Hoppe-Seyler*, 375(2):89–92.

[Wang et al., 2008] Wang, Q. F., Hou, Y. H., Xu, Z., Miao, J. L., and Li, G. Y. (2008). Purification and properties of an extracellular cold-active protease from the psychrophilic bacterium Pseudoalteromonas sp. NJ276. *Biochemical Engineering Journal*, 38(3).

[Wang et al., 2005] Wang, Q. F., Miao, J. L., Hou, Y. H., Ding, Y., Wang, G. D., and Li, G. Y. (2005). Purification and characterization of an extracellular cold-active serine protease from the psychrophilic bacterium Colwellia sp. NJ341. *Biotechnology Letters*, 27(16):1195–1198.
